# Progress in Advanced Infrared Optoelectronic Sensors

**DOI:** 10.3390/nano14100845

**Published:** 2024-05-12

**Authors:** Xiang Yu, Yun Ji, Xinyi Shen, Xiaoyun Le

**Affiliations:** 1 School of Physics, Beihang University, Beijing 100191, China; 2 Beijing Advanced Innovation Center for Big Data-Based Precision Medicine, School of Medicine and Engineering, Beihang University, Beijing 100191, China; 3 Beijing Key Laboratory of Advanced Nuclear Energy Materials and Physics, Beihang University, Beijing 100191, China; 4 Department of Electrical and Computer Engineering, National University of Singapore, 4 Engineering Drive 3, Singapore 117583, Singapore

**Keywords:** infrared optoelectronic sensor, photovoltaic effect, responsivity, 2D materials, image sensing

## Abstract

Infrared optoelectronic sensors have attracted considerable research interest over the past few decades due to their wide-ranging applications in military, healthcare, environmental monitoring, industrial inspection, and human–computer interaction systems. A comprehensive understanding of infrared optoelectronic sensors is of great importance for achieving their future optimization. This paper comprehensively reviews the recent advancements in infrared optoelectronic sensors. Firstly, their working mechanisms are elucidated. Then, the key metrics for evaluating an infrared optoelectronic sensor are introduced. Subsequently, an overview of promising materials and nanostructures for high-performance infrared optoelectronic sensors, along with the performances of state-of-the-art devices, is presented. Finally, the challenges facing infrared optoelectronic sensors are posed, and some perspectives for the optimization of infrared optoelectronic sensors are discussed, thereby paving the way for the development of future infrared optoelectronic sensors.

## 1. Introduction

The infrared region of the electromagnetic spectrum, spanning near-infrared regime (0.78–2.5 μm), mid-infrared regime (2.5–25 μm), and far-infrared regime (25–1000 μm), is of significant interest due to its wide applications in optical communication, health monitoring, industrial inspection, environment monitoring, and human–computer interaction systems [1]. Infrared optoelectronic sensors, which are able to selectively convert infrared photons into electrical signals, play a crucial role in advancing the utilization of infrared light [2,3]. According to their energy conversion processes, infrared optoelectronic sensors can be broadly classified into photon-type, photothermal-type, and hybrid-type devices. Photon-type optoelectronic sensors utilize photosensitive semiconductors to absorb incident infrared photons, exciting bound-state electrons to the conduction band of the photosensitive semiconductor to generate photogenerated electron–hole pairs. These electron–hole pairs are then separated and transported under external or built-in electric fields to form electrical signals, achieving a direct conversion of light energy to electricity. In contrast, photothermal-type infrared optoelectronic sensors first absorb infrared photons to generate thermal energy through the photothermal effect. Then, that thermal energy induces temporal or spatial variations in the temperature of the sensors, driving carrier migration for electrical signal generation, thereby enabling indirect conversion of the light energy to electricity. Hybrid-type infrared optoelectronic sensors leverage infrared photons to simultaneously generate electron–hole pairs and induce temperature variations for measurable electrical signals. Commercialized infrared optoelectronic sensors primarily rely on epitaxially grown crystalline inorganic III–V compound semiconductors [4], which are insufficient to meet the ever-changing demands of various applications. Hence, a variety of photosensitive materials and nanostructures have been developed to construct diverse infrared optoelectronic sensors. These photosensitive materials and nanostructures include narrow-bandgap two-dimensional (2D) semiconductor materials with high carrier mobility and absorption coefficients [5,6], a narrow-bandgap conjugated polymer [7,8], ferroelectric materials with high pyroelectric coefficients [9,10], and homo-/heterojunction structures with a strong built-in electrical field for carrier separation and transport [11,12,13,14]. With diverse functional materials and device configurations, the performance metrics of infrared optoelectronic sensors, including their response range, responsivity, detectivity, and response speed, can vary significantly, tailoring them to specific demands. The rapid development of infrared optoelectronic sensors, together with the daily evolution and industrial requirements of infrared applications, highlights the critical need for a comprehensive understanding of these sensors.

This paper provides a comprehensive review of recent advancements in infrared optoelectronic sensors. It begins by elucidating their working mechanisms, followed by an introduction to the key metrics utilized to evaluate device performance. Next, it offers an overview of promising materials and nanostructures for high-performance infrared optoelectronic sensors, along with the room-temperature performance of state-of-the-art devices. Moreover, it outlines the recent applications of infrared optoelectronic sensors. Finally, it discusses the challenges and prospects facing infrared optoelectronic sensors, providing guidance for the development of future infrared optoelectronic sensors.

## 2. Working Mechanisms

### 2.1. Photovoltaic Effect

The photovoltaic effect refers to the phenomenon by which photoexcited electron–hole pairs are generated, separated, and transported under the driving force of internal electric fields to produce electric signals [15]. Based on the photovoltaic effect, infrared optoelectronic sensors absorb light to generate electron–hole pairs, which are then extracted and accelerated by internal electric fields, resulting in sizable photocurrent/photovoltaic signals. Their internal electric fields mainly arise from the formation of a depletion region at the interface of Schottky junctions [16,17], semiconducting homojunctions/heterojunctions [18,19,20,21], and semiconductor/electrolyte junctions [22,23].

**Figure 1:**
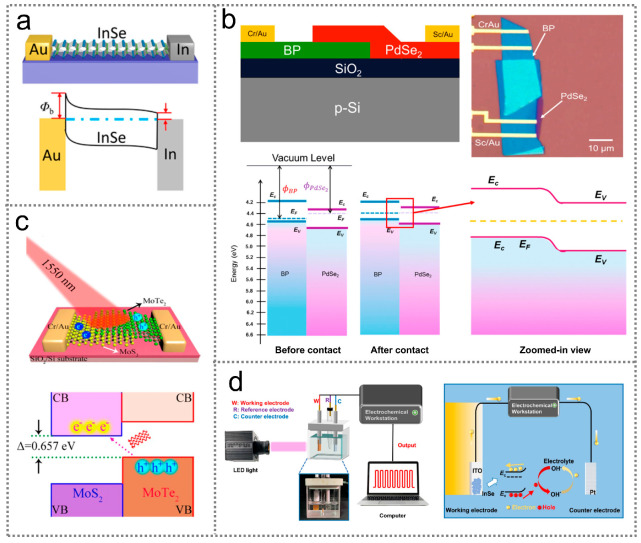
Working mechanisms of photon-type infrared optoelectronic sensors. (**a**) Working mechanism of an infrared optoelectronic sensor based on Schottky junctions [24] (with permission from the American Chemical Society, 2018). (**b**) Working mechanism of an infrared optoelectronic sensor based on a p-n junction with intralayer transition [25] (with permission from the American Chemical Society, 2020). (**c**) Working mechanism of an infrared optoelectronic sensor based on a p-n junction with interlayer transition [26] (with permission from the American Chemical Society, 2016). (**d**) Working mechanism of an infrared optoelectronic sensor based on a semiconductor/electrolyte junction [23] (with permission from the American Chemical Society, 2022).

The energy difference between the Fermi levels of metals and semiconductors causes a large potential difference, forming Schottky barrier junctions at metal/semiconductor interfaces. The mechanism of electrical signal generation in Schottky junctions can be understood with the assistance of energy band diagrams. Figure 1a illustrates the energy band diagram of a typical Schottky junction formed at a Au/InSe interface [24]. Due to the Fermi level difference between Au and InSe, electrons in the InSe migrate toward the Au electrode, leaving behind positively charged ions on the InSe side. Consequently, the energy bands of the InSe are bent upward to form a Schottky junction at the Au/InSe interface. Upon illumination, photoexcited electron–hole pairs can be separated and extracted by the Schottky junction, generating electrical signals. 

A p-n junction is formed due to the diffusion of carriers under the driving force of the carrier concentration difference in p-type and n-type semiconductors. For example, when p-type black phosphorous (BP) and n-type PdSe_2_ come into contact, the holes in BP and electrons in PdSe_2_ diffuse in opposite directions, establishing a built-in electric field at the p-BP/n-PdSe_2_ interface, as shown in Figure 1b [25]. Upon illumination, electrons in the valence band of BP can be excited to the conduction band, generating photogenerated electron–hole pairs. Subsequently, photogenerated electron–hole pairs near the BP/PdSe_2_ interface can diffuse to the p-n junction, where they are separated and swept toward opposite directions by the built-in electric field, forming electrical signals in the external circuit. Infrared optoelectronic sensors based on p-n junctions can generate photogenerated electron–hole pairs based on two main approaches: the band-to-band transition of electrons within an individual semiconductor (Figure 1b), and the interlayer transition of electrons at the interface of type-II staggered semiconductors. Figure 1c presents a representative energy band diagram of interlayer electron transition based on a type-II MoTe_2_/MoS_2_ heterostructure [26]. Under illumination, the electrons in the valence band of the MoTe_2_ can be excited to the conduction band of the MoS_2_, thereby creating electrical signals. Due to the small energy offset (0.657 eV) between the valence band of the MoTe_2_ and the conduction band of the MoS_2_, an infrared response is beyond the limits of the intrinsic bandgaps of the MoS_2_ and MoTe_2_ materials. 

A semiconductor/electrolyte junction is established because of the disparity between the work functions of semiconductors and redox potential electrolytes. Figure 1d displays the working mechanism of a representative infrared optoelectronic sensor based on a semiconductor/electrolyte junction, where InSe is contact with the electrolyte [23]. Since the work function of InSe is smaller than the redox potential of the electrolyte, electrons in the InSe flow into the electrolyte until an electronic equilibrium is achieved. As a consequence, a built-in electric field is established and works similar to a Schottky junction. Upon illumination, the photogenerated electron–hole pairs generated in the InSe are separated and transferred under the driving force of the built-in electric field, with electrons and holes moving toward the Pt and ITO electrodes, respectively. Therefore, light-induced electrical signals are created in the external circuit. 

### 2.2. Photoconductive Effect

The photoconductive effect is an effect that means the conductivity of semiconductors changes with the incident light’s intensity. The generation of an electric signal in infrared optoelectronic sensors based on the photoconductive effect is quite similar to that based on the photovoltaic effect, wherein photogenerated electron–hole pairs are created by absorbing photons. However, in photoconductive sensors, the separation and transport of photogenerated electron–hole pairs require an external electric field as the driving force [27,28]. 

### 2.3. Pyroelectric Effect

**Figure 2:**
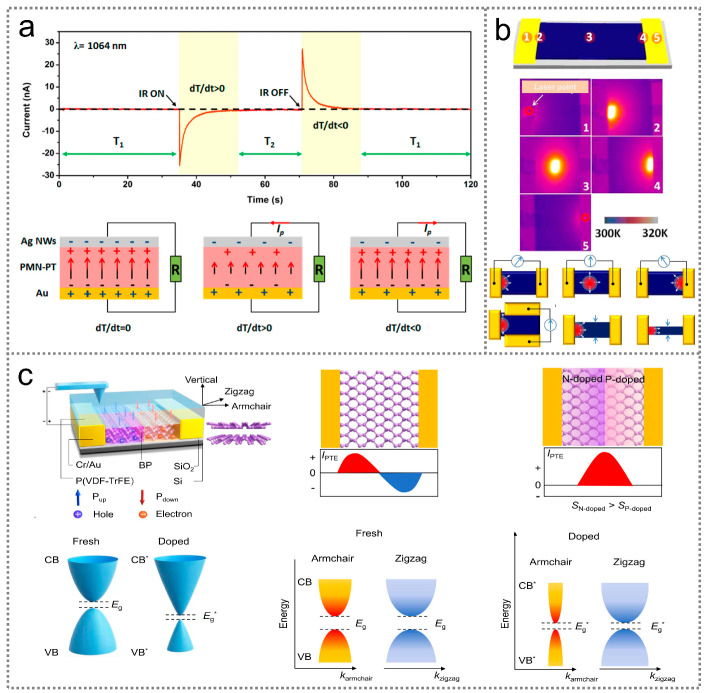
Working mechanism of photothermal-type infrared optoelectronic sensors. (**a**) Working mechanism of an infrared optoelectronic sensor based on the pyroelectric effect [33] (with permission from the American Chemical Society, 2016). (**b**) Working mechanism of an infrared optoelectronic sensor on the basis of the photothermoelectric effect in a single semiconductor [37] (with permission from the American Chemical Society, 2015). (**c**) Working mechanism of an infrared optoelectronic sensor based on the photothermoelectric effect in a p-n semiconducting homojunction. CB, VB, and *E*g stand for the conduction band, valence band and bandgap of pristine BP, respectively. CB*, VB*, and *E*g* are the conduction band, valence band, and bandgap of doped BP [40] (with permission from Springer Nature, 2022).

The pyroelectric effect refers to the phenomenon in which electric charges are generated in response to a change in spontaneous polarization caused by a temperature variation, which typically occurs in certain polar materials [29]. Pyroelectric infrared optoelectronic sensors have several advantages over other types of sensors, such as room-temperature operation, a wide wavelength response, and low cost, enabling their use for various applications [30,31,32]. To elucidate the working mechanism of pyroelectric infrared optoelectronic sensors, the process of electrical signal generation in a device utilizing a ferroelectric PMN-PT layer as its pyroelectric component is introduced. Figure 2a exhibits the schematic diagram and output current of a PMN-PT pyroelectric infrared optoelectronic sensor, where the PMN-PT layer is sandwiched between the top Ag nanowire electrode and the bottom Au electrode [33]. Under dark conditions (d*T*/d*t* = 0), the electric dipoles within the PMN-PT layer are well aligned and oscillate around their aligned axes to a certain degree, resulting in a stable spontaneous polarization strength. As a consequence, negative and positive charges are attracted to the Ag nanowire electrode and the Au electrode, respectively, reaching an equilibrium state at which no current can be detected in the external circuit. Upon illumination, the temperature of the PMN-PT sensor increases due to photothermal effect (d*T*/d*t* > 0), intensifying the oscillation of the electric dipoles and reducing the spontaneous polarization strength in the PMN-PT. Hence, the attracted negative and positive charges move toward the Au electrode and the Ag nanowire electrode, respectively, until a new thermal equilibrium state is established. Consequently, a negative current signal is observed in the external circuit. When the light is removed, the temperature of the sensor gradually decreases to its original state (d*T*/d*t* < 0), which suppresses the oscillation of electric dipoles in the PMN-PT, leading to a higher average spontaneous polarization strength. Consequently, a positive current signal is generated in the external circuit. This light-induced pyroelectric current can be obtained using the following equation [34]:

(1) $$I_{\mathrm{pyro}}=\frac{\eta p_{C}SP}{cdA}$$

where *η* stands for the emissivity of the illuminated device surface, *P* is the incident light’s intensity, *d* is the device thickness, *P*_c_ represents the pyroelectric coefficient, *c* stands for the specific heat capacity, and *A* is a parameter related to the device’s thermal time constant. For years, the pyroelectric effect has been considered to only exist in non-centrosymmetric materials, and always exhibited a sharp decay away from the phase transition temperature, thereby limiting its working temperature. In 2020, the pyroelectric effect was demonstrated in interfaces with polar symmetry, which exhibit a weak temperature dependence, suitable for utilization across a wide temperature range [35].

### 2.4. Photothermoelectric Effect

The photothermoelectric effect leverages the coupling of the photothermal effect and thermoelectric effect in semiconductors to generate electric potential [36]. Figure 2b shows a typical photothermoelectric infrared optoelectronic sensor which possesses a planar device configuration with electrodes positioned at both ends of a semiconductor [37]. When localized illumination is applied to the sensor, a temperature gradient Δ*T* within the semiconductor is established due to the nonuniform heating caused by the incident light. The temperature gradient Δ*T* drives charge carriers to diffuse from the hotter side to the colder side of the semiconductor. Consequently, an electric potential difference Δ*V* is produced owing to the Seebeck effect. The relationship between the electric potential difference Δ*V* and the temperature gradient Δ*T* can be expressed utilizing the following formula [38]:

(2) ΔV=SΔT

where *S* represents the Seebeck coefficient of the semiconductor. The Seebeck coefficient can be described by the Mott equation [39]:

(3) $$S=-\frac{\pi^{2}k_{B}^{2}T}{3e}{\left.\left(\frac{d\left(\ln\sigma \right)}{dE}\right)\right|}_{E=E_{f}}$$

where *k*_B_ stands for the Boltzmann constant, *T* represents the absolute temperature, *e* is the elementary charge, *σ* is the electrical conductivity, and *E*_f_ stands for the Fermi level. According to this equation, semiconductor materials with high electrical conductivity, a high light absorption coefficient, low thermal conductivity, and a low heat capacity can contribute to high-performance photoelectric infrared optoelectronic sensors. 

Besides n-type or p-type semiconductors, photothermoelectric infrared optoelectronic sensors can also be constructed by utilizing the p-n junctions in certain materials. As illustrated in Figure 2c, a p-n homojunction in a BP material can be achieved based on electrical doping [40]. Under illumination, the electrons and holes in the homojunction are driven by the temperature gradient and transported toward opposite directions, resulting in enhanced current signals on both sides. Since the fundamental driving force for charge carrier diffusion is the temperature gradient-induced carrier concentration gradient, highly sensitive thermoelectric infrared optoelectronic sensors can be realized with device configurations that allow for larger temperature gradients.

### 2.5. Others

**Figure 3:**
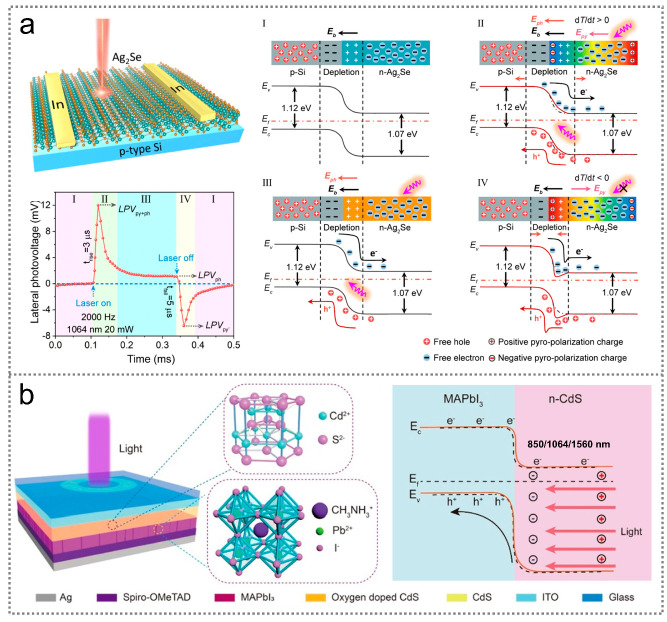
The working mechanism of infrared optoelectronic sensors based on coupling effects. (**a**) Working mechanism of an infrared optoelectronic sensor based on the pyroelectric–photovoltaic effect [42] (with permission from the American Chemical Society, 2022). (**b**) Working mechanism of an infrared optoelectronic sensor based on the pyroelectric–photothermoelectric effect [43] (with permission from the American Chemical Society, 2023).

Taking the advantages of photovoltaic and photothermal effects, infrared optoelectronic sensors can be constructed based on the pyroelectric–photovoltaic effect, which is a coupling of pyroelectric polarization, semiconductor/ferroelectrics characteristics, and photoexcitation processes [41]. Figure 3a illustrates the working mechanism of a pyroelectric–photovoltaic infrared optoelectronic sensor, which is constructed based on a p-Si/n-Ag_2_Se heterojunction [42]. Upon light illumination, photogenerated electrons and holes move toward the Ag_2_Se and Si sides due to the photovoltaic effect, respectively. As a result, a positive electric signal is produced in the external circuit. Meanwhile, a rapid transient increase in the temperature is achieved in the Ag_2_Se because of the photothermal effect, leading to a positive pyroelectric signal traversing the Ag_2_Se. Since the light-induced photovoltaic signal and pyroelectric signal possess the same polarity, the electrical signal of a pyroelectric–photovoltaic infrared optoelectronic sensor can achieve enhanced infrared responses compared to infrared optoelectronic sensors based on the pyroelectric effect or photovoltaic effect alone. 

The pyroelectric–photothermoelectric effect is another vital mechanism for constructing infrared optoelectronic sensors because it maximizes the light-induced heat to generate electrical signals. Figure 3b illustrates the working mechanism of a pyroelectric–photothermoelectric infrared optoelectronic sensor, where a heterostructure composed of CH_3_NH_3_PbI_3_ (MAPbI_3_) and CdS materials serves as the photosensitive component [43]. Under illumination, the temperature of the sensor gradually increases due to the photothermal effect, leading to temporal variations in the device temperature for pyroelectric signals. Moreover, the photothermal effect can create a longitudinal temperature gradient in the MAPbI_3_/CdS heterojunction, thereby resulting in photothermoelectric signals that align with the pyroelectric signals. The combination of the pyroelectric and photothermoelectric effects is promising for achieving high responsivity.

## 3. Key Performance Metrics

The critical parameters for evaluating the performance of infrared optoelectronic sensors include their spectral response range, responsivity *R*, response speed, gain *G*, Noise-equivalent power NEP, specific detectivity D*, on/off ratio *R*_ratio_, linear dynamic range LER, and external quantum efficiency EQE. 

The spectral response range refers to the region of wavelength that the infrared optoelectronic sensors can detect. Each infrared optoelectronic sensor can only respond to a specific wavelength due to the limitations imposed by their photosensitive material’s properties, working mechanisms, and device configurations. 

Responsivity *R* is one the most important device parameters for infrared optoelectronic sensors, as it represents the magnitude of the photocurrent produced by illumination at a given light intensity and wavelength. Responsivity *R* can be defined as

(4) $$R=\frac{I_{\mathrm{ph}}}{P_{\mathrm{in}}}$$

where *I*_ph_ is the light-induced current density (A cm^−2^) of the infrared optoelectronic sensor and *P*_in_ stands for the incident light power density (W cm^−2^).

Response speed can be evaluated by the response time and recovery time. Generally, the response time and recovery time are defined as the time required for the photocurrent/photovoltage to increase from 10% to 90% and to decrease from 90% to 10%, respectively. The response speed relies on many factors, especially material properties, interface defects, and temperature.

Gain *G* reflects the magnitude of recycled photoexcitation based on the accumulation mechanism, which is determined by the lifetime of excitons. Gain *G* can be expressed using the formula

(5) $$G=\frac{\tau_{\mathrm{life}}}{\tau_{\mathrm{transit}}}=\frac{\tau_{\mathrm{life}}}{L^{2}/\mu V}$$

where *τ*_life_ stands for the lifetime of photogenerated excitons and *τ*_transit_ means the time taken for photogenerated excitons to transit through a channel. *L* represents the channel length, *μ* is the carrier mobility, and *V* is the voltage exerted on the channel. In a photovoltaic optoelectronic infrared sensor, the gain is equal to 1 unless carrier multiplication effects are involved. However, in a photoconductive optoelectronic infrared sensor, one type of carrier (typically holes) is captured in trap states, while the other type of carrier (electrons) traverses the channel. If the lifetime *τ*_life_ of the holes is larger than the transit time *τ*_transit_ of the electrons, the electrons can recirculate many times before recombining with the captured holes, resulting in a gain greater than 1. In this case, increasing the trapping of holes can lead to a higher gain [44,45].

Noise-equivalent power (NEP) is the minimum light power required to distinguish a signal from noise in an optoelectronic sensor. The NEP can be utilized to evaluate the sensitivity of the device and is defined using the equation

(6) $$\mathrm{NEP}=\left(\frac{I_{n}}{\Delta f^{1/2}R}\right)$$

where *I*_n_ stands for the noise current detected in dark conditions and Δ*f* represents the electrical bandwidth of the noise measurement. 

The specific detectivity D* reflects the sensitivity of the optoelectronic sensors to weak light detection and is given by the following formula:

(7) $$D^{*}=\frac{A^{1/2}R}{\mathrm{NEP}}=\frac{R{\left(A\Delta f\right)}^{1/2}}{I_{n}}$$

where *A* is the effective device area. The unit of specific detectivity D* is Jones (1 Jones = 1 cm Hz^1/2^ W^−1^).

The on/off ratio *R*_ratio_ is the ratio between the dark noise current and the photocurrent and can be calculated by the formula

(8) $$R_{\mathrm{ratio}}=\frac{I_{\mathrm{ph}}}{I_{n}}$$

The linear dynamic range LDR is the specific range in which the photocurrent shows a linear relationship to the incident light power. The linear dynamic range LDR is described by the following equation:

(9) $$\mathrm{LDR}=20\log\left(\frac{P_{\max}}{P_{\min}}\right)=20\log\left(\frac{J_{\max}}{J_{\min}}\right)$$

where *P*_max_ stands for the maximum incident light power beyond which the photoresponses deviate from their linear region and *P*_min_ represents the minimum detectable light density. *J*_max_ and *J*_min_ are the maximum and minimum values of photocurrent density, respectively.

The external quantum efficiency EQE depends on the number of primary charge carriers generated per single input photon, which plays an important role in determining responsivity. External quantum efficiency EQE can be calculated using the formula

(10) $$\mathrm{EQE}=\frac{I_{\mathrm{ph}}h\nu }{P_{\mathrm{in}}e}$$

where *hν* represents the photon’s energy. 

## 4. Materials and Their Performances

### 4.1. 2D Semimetals and Semiconductors

Two-dimensional materials hold great potential for building highly integrated and efficient infrared optoelectronic sensors due to their thickness-tunable bandgaps, high carrier mobility, and strong optical absorption. Because their infrared range corresponds to a low photon energy of about 1.55 eV, most of the 2D materials used in infrared optoelectronic sensing devices are semimetals and narrow-bandgap semiconductors. Semimetals, such as graphene, TaAs, PdTe_2_, WTe_2_, and TaIrTe [46,47,48], possess gapless electronic band structures with linear cones, enabling broadband infrared sensing extending to the far-infrared spectral region [49]. The lifetime of the photogenerated carriers in semimetals is dramatically decreased via fast electron–electron scattering, thereby allowing for rapid responses [50]. However, because of their gapless nature, infrared optoelectronic sensors based on semimetals usually suffer from high dark currents. Narrow-bandgap 2D semiconductors, including BP [51], black AsP (B-AsP) [52], Bi_2_O_2_Se [53], tellurene [54], metal chalcogenides, and transition-metal dichalcogenides [55], exhibit thickness-tunable bandgaps, which contribute to their low dark currents. Table 1 summarizes the bandgaps of promising 2D materials for infrared sensing. Among these 2D materials, graphene, BP, and metal chalcogenides are most frequently utilized to fabricate infrared optoelectronic sensors.

**Figure 4:**
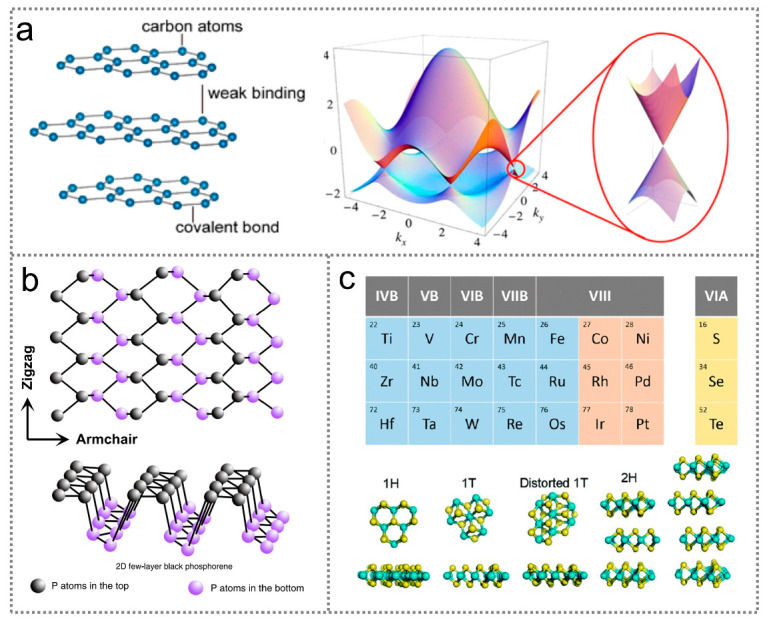
Two-dimensional materials for infrared optoelectronic sensors. (**a**) Atomic structure and band structure of graphene [57] (with permission from the American Chemical Society, 2018). (**b**) Atomic structure of BP [64] (with permission from Springer Nature, 2018). (**c**) Elements for transition metal chalcogenides, and structures of transition metal chalcogenides [78] (with permission from the American Chemical Society, 2021).

Graphene, first mechanically exfoliated from graphite in 2004 [56], has become popular due to its intriguing electronic and optical properties. Graphene possesses an in-plane chemical bond connecting its carbon atoms and stacks through the van der Waals forces between its layers. In graphene, a single layer of carbon atoms with sp^2^ hybridization arranged in a honeycomb lattice, with each carbon atom in-plane bonded to its three nearest carbon atoms, as illustrated in Figure 4a [57]. Graphene is gapless and dispersed linearly near the Dirac point, making it theoretically capable of responding to all photons. This property offers advantages for wideband photodetection, spanning from the ultraviolet to the terahertz spectral regimes [58]. The conductivity and mobility of graphene are mainly determined by its defects and are nearly independent of temperature [59]. With high carrier mobility (up to 2 × 10^5^ cm^2^ V^−1^ s^−1^) at room temperature [60], graphene enables ultrafast infrared sensing based on photovoltaic, photothermoelectric, and photoconductive effects.

BP is a direct-bandgap semiconductor with a thickness-tunable bandgap ranging from 0.3 eV to 2.0 eV. Since its rediscovery in 2014, BP has emerged as a preferred candidate for infrared optoelectronic sensing due to its unique characteristics, including its strong intralayer anisotropy, high mobility (10^3^ cm^2^ V^−1^ s^−1^), and strong optical absorption [61,62,63]. Bulk BP possesses an orthorhombic structure with a D_2h_^18^ space group symmetry. Within a single atomic layer of BP, each phosphorous atom bonds with three neighboring atoms, resulting in two distinct directions: the armchair direction along its x axis, and the zigzag direction along its y axis, as illustrated in Figure 4b [64]. The highly anisotropic arrangement of phosphorous atoms results in anisotropic electric band dispersion, thereby leading to anisotropic optoelectronic properties. Benefiting from its moderate bandgap, large tunability, and anisotropy, few-layer BP holds significant promise for polarized infrared optoelectronic sensors. Due to its noncentrosymmetric structure, BP is expected to exhibit pyroelectricity. In addition, the bandgap of BP demonstrates anomalous strain dependence because of its puckered lattice structure, enabling the continuous and reversible tuning of its operating wavelengths during infrared optoelectronic sensing through strain modulation. With its combination of a narrow bandgap, high charge carrier concentration, and noncentrosymmetric structure, BP enables infrared optoelectronic sensing based on photovoltaic, photothermoelectric, and pyroelectric effects [65]. 

Non-transition metal chalcogenides with narrow and tunable bandgaps are emerging as popular candidates for the construction of infrared optoelectronic sensors. These materials possess chemical formulas of MX, MX_2_, or M_2_X_3_, where M can be In, Ge, Bi, Sb, Pt, or Sn, and X can be S, Se, or Te. Among the non-transition metal chalcogenides, In-based narrow-bandgap materials such as InSe, α-In_2_Se_3_, and β-In_2_Se_3_ are notable. InSe exhibits a direct bandgap of 1.26 eV in bulk, and an indirect bandgap of 2.72 eV in monolayer [66]. Due to its small electron effective mass, InSe has a high electron mobility of about 10^3^ cm^2^ V^−1^ S^−1^ and a low electron–hole recombination rate, enabling a high optoelectronic response. Surface doping using AuCl_3_ can modify the energy band structure of InSe, which facilitates the separation of photogenerated electron–hole pairs, leading to an improved optoelectronic sensing performance [67]. Two-dimensional α-In_2_Se_3_ with a noncentrosymmetric crystal structure exhibits robust ferroelectricity in both in-plane and out-of-plane directions, even when reduced to a monolayer [68]. The bandgap of α-In_2_Se_3_ shows a strong thickness dependence, which is 1.3 eV for bulk [69]. The ferroelectricity and direct bandgap of α-In_2_Se_3_ make it well suited for applications in infrared optoelectronics. β-In_2_Se_3_ is predicted to be an indirect-bandgap semiconductor at all thicknesses, with bandgap changes from 1.45 eV (bulk) to 1.5 eV (6.2 nm in thick) [70]. GeSe has a distorted NaCl-type crystalline structure, with an indirect bandgap of 1.10 eV in bulk and a direct bandgap of 1.87 eV in monolayer [71]. The detection wavelength of a GeSe-based optoelectronic sensor is limited to about 1 µm due to its interband transitions, which can be broadened to 1.6 µm by introducing Ge vacancies [72]. The Sn-based narrow-bandgap metal chalcogenides include SnS, SnSe, and SnSe_2_. Orthorhombic SnS is an anisotropic layered semiconductor. It possesses a mid-infrared direct bandgap of 1.19 eV and an absorption coefficient higher than 10^4^ cm^−1^ [73]. SnSe has an orthorhombic lattice configuration, maintaining an indirect bandgap while changing its thickness from bulk (0.89 eV) to monolayer (1.63 eV) [70]. SnSe has a high optical absorption coefficient of about 10^5^ cm^−1^, which is about 1–2 orders of magnitude larger than that of silicon and GaAs [74]. Besides its attractive bandgap and absorption characteristics, SnSe also generates a recorded thermoelectric figure-of-merit of about 2.6 from its anharmonicity, making it well-suited for constructing infrared photothermoelectric optoelectronic sensors [75]. SnSe_2_ has an indirect bandgap that changes from 1.2 eV (bulk) to 2.04 eV (monolayer) [70]. Theoretical calculation results show that the valence band and conduction band of SnSe_2_ reside at a point along the line G m and the M point, respectively. When the thickness of SnSe_2_ is reduced to a monolayer, its valence band becomes flat, increasing the density of tis states. The density of the states of monolayer and bilayer SnSe_2_ exhibit van Hove singularities [70]. These characteristics enable SnSe_2_’s high photoabsorption. Tetradymites are an important class of non-transition metal chalcogenides for infrared optoelectronic sensing and include Bi_2_S_3_, Bi_2_Se_3_, Bi_2_Te_3_, Sb_2_Se_3_, and Sb_2_Te_3_. Bi_2_S_3_, Bi_2_Se_3_, Bi_2_Te_3_, and Sb_2_Te_3_ possess direct bandgaps of 1.3 eV, 0.35 eV, 0.21 eV, and 0.45 eV in bulk, making them ideal for absorbing infrared photons. Moreover, Bi_2_Se_3_, Bi_2_Te_3_, and Sb_2_Te_3_ also exhibit outstanding thermoelectric properties owing to their high electron mobility induced by a strong spin-orbit [76,77]. The combination of a narrow bandgap and high thermoelectric performance makes tetradymites promising for infrared sensing applications.

Transition metal chalcogenides share the same chemical formula as non-transition metal chalcogenides. However, in transition metal chalcogenides, the M stands for transition metals, such as Mo, W, Hf, and Zr (Figure 4c) [78]. Transition metal chalcogenides can exist in various structures, including 1H, 1T, distorted 1T, 2H, and 3R, where 1T, 2H, and 3R are the most stable and common. The conductivity of transition metal chalcogenides varies greatly with their composition, exhibiting semiconducting or metallic properties. Semiconducting transition metal chalcogenides undergo a transition from an indirect bandgap to a direct bandgap as their thickness decreases to monolayer or bilayer. The bandgaps of typical semiconducting transition metal chalcogenides are in the range of 0.21–2.1 eV [78,79], which can be modulated through strain engineering, electric fields, temperature, and alloying to meet specific demands. For example, DFT calculations predict that the bandgap of monolayer transition metal chalcogenides can be adjusted from 0.68 eV to 2.34 eV through a small tensile strain of 8% [80]. Furthermore, strain can induce an indirect-to-direct bandgap transition in monolayer ZrS_3_ and HfS_3_. Transition metal dichalcogenides, including MoS_2_, MoSe_2_, MoTe_2_, WS_2_, WSe_2_, and MoTe_2_, are popular materials for infrared sensing because their monolayers exhibit strong light–matter interactions [78]. MoS_2_ is one of the most popular transition metal chalcogenides for infrared sensing, with a bandgap ranging from 1.2 eV to 1.9 eV, corresponding to a photodetection of wavelengths of about 0.652–1.033 μm. Experimental results indicate that monolayer MoS_2_ possesses high-field-effect electron and hole mobilities of about 260 cm^2^ V^−1^ s^−1^ and 175 cm^2^ V^−1^ s^−1^ [81], respectively. This facilitates fast infrared photodetection.

**Table 1:** Bandgaps of promising 2D semiconductors for infrared optoelectronic sensors.

Material	Bandgap (eV)		Ref.
	Bulk	Monolayer	
Graphene	0	0	[60]
Tellurene	0.325	1.265	[82]
BP	0.3	2.0	[83]
B-AsP	0.3	0.92	[84,85]
InSe	1.26	2.72	[66]
α-In_2_Se_3_	1.3	1.55	[86]
β-In_2_Se_3_	0.7	1.29	[87]
SnS	1.19	2.46	[73]
SnSe	0.89	1.63	[70]
SnSe_2_	1.2	2.04	[72]
Bi_2_S_3_	1.3	-	[70]
Bi_2_Se_3_	0.35	-	
Bi_2_Te_3_	0.21	-	
Sb_2_Te_3_	0.45	-	
ZrSe_3_	0.75	1.17	
GeSe	1.1	1.87	[71]
TiS_3_	1.02	1.06	[79]
TiSe_3_	0.21	0.57	
MoS_2_	1.2	1.9	[81]
MoSe_2_	1.1	1.44	[88,89]
MoTe_2_	0.88	0.90	[90,91,92,93]
WS_2_	1.3	2.05	[89,94,95,96]
WSe_2_	1.2	1.70	[97,98]
WTe_2_	0.7–0.81	0.18	[78]
ReSe_2_	1.09	1.24	[99]
PdSe_2_	0.03	1.43	[100]
Bi_2_O_2_Se	0.8	-	[101]

In addition to the aforementioned 2D materials, transition metal chalcogenophosphates such as FePS_3_, FePSe_3_, Mn_2_P_2_X_3_, and Ni_2_P_2_Te_3_, and Bi-based oxychalcogenides such as Bi_2_O_2_Se, are also promising candidates for constructing infrared optoelectronic sensors. Furthermore, 2D materials can be utilized in various heterostructures to effectively generate and separate photogenerated electron–hole pairs for infrared sensing. Heterostructures intended for interlayer exciton generation have attracted considerable attention because they break the limits of intrinsic bandgaps. Their interlayer excitons exhibit a lower-energy spectral peak with a much weaker intensity compared to intralayer excitons, granting them a high photoresponse. The performances of advanced infrared optoelectronic sensors based on 2D materials are presented in Table 2.

### 4.2. Group III–V Semiconductor Materials

Group III–V semiconductors with a narrow bandgap have been widely utilized in constructing infrared optoelectronic sensors due to their high carrier mobility, excellent stability, low dielectric constants, and high absorption coefficients [145]. So far, group III–V semiconductor quantum dots, thin films, and single-crystal nanowires have been developed to achieve high-performance infrared sensing. Infrared sensors based on group III–V semiconductor quantum dots and thin films usually exhibit relatively poor performance compared to those based on single-crystal nanowires, because many bulk and surface defects are created in quantum dots and thin films during their fabrication process. Semiconductor single-crystal nanowires can transport charge carriers along their axis, reducing carrier scattering and trapping, thereby promoting device performance [146]. Chemical vapor deposition (CVD) is one of the most effective approaches to synthesize high-quality group III–V semiconductor nanowires. For example, InAs nanowires fabricated on Si substrates utilizing the CVD method show an ultra-high carrier mobility of 10^4^ cm^2^ V^−1^ s^−1^ and an attractive narrow bandgap of about 0.354 eV [147]. In_x_Ga_1−x_As nanowires prepared by alloying InAs and GaAs materials exhibit a tunable bandgap (0.35–1.42 eV), meeting their sensors’ ever-changing requirements [148]. In particular, In_0.65_Ga_0.35_As nanowire materials exhibit a wide spectral response up to 2.0 μm. The light absorption in group III–V semiconductor nanowires such as GaSb and InP nanowires exhibits anisotropy, making them promising for detecting the polarization properties of incident infrared light [149,150]. 

Since heterojunctions provide a powerful built-in electric field for carrier separation and transport, group III–V single-crystal nanowires have been designed into various heterojunctions, including vertically aligned nanowire homojunctions/heterojunctions, core–shell nanowire heterojunctions, and superlattice structures. For example, a p-i-n homojunction based on InGaAs demonstrates a spectral response from 1.2 μm to 1.7 μm, enabling high-speed optical data reception (32 Gb s^−1^) [151]. Additionally, a p-n GaAs_1–x_Sb_x_/InAs core–shell heterojunction nanowire demonstrates a wide spectral response ranging from 0.488 μm to 1.8 μm, suitable for wide-spectrum photodetection [152]. Infrared optoelectronic sensors based on group III–V semiconductor type-II superlattices have gained significant interest due to their broad-spectrum response, low noise, and high sensitivity. For instance, the GaN/AlN superlattices embedded in GaN nanowires rely on the transitions between quantum-confined electron levels in semiconductor heterostructures, enabling the absorption of light up to 1.63 μm [153]. This structure holds promise for ultrafast and ultrasensitive infrared sensing. Infrared sensors based on various group III–V semiconductors and device structures demonstrate distinct performances, as summarized in Table 3.

### 4.3. Ferroelectric Materials

Ferroelectric materials are a key group for the fabrication of infrared optoelectronic sensors due to their remarkable pyroelectric effect and anomalous photovoltaic effect. Compared with other types of pyroelectric materials, ferroelectric materials possess unique advantages, such as high pyroelectric coefficients, excellent chemical and mechanical stability, and low manufacture costs [169]. Moreover, some ferroelectric materials possess a narrow bandgap, making them suitable for simultaneously utilizing pyroelectric and photovoltaic effects to detect infrared light. Ferroelectric LiTaO_3_ single-crystal materials possess a high Curie temperature, excellent stability, and a high pyroelectric coefficient, making them benchmark materials for infrared sensing applications [170]. Single-crystal materials based on a lead magnesium niobate–lead titanate solid solution system, xPbMg_1/3_Nb_2/3_O_3_-(1−x)PbTiO_3_ (PMN-PT), have been developed as promising candidates for detecting infrared light. The high pyroelectric coefficient (≥500 μC m^−2^ K^−1^) of PMN-PT materials was first presented by Davis et al. [171]. Since then, many efforts have been made to develop PMN-PT infrared optoelectronic sensors. The pyroelectric coefficient of a PMN-PT single crystal with a PMN:PT molar ratio of 72:28 is 7.5 × 10^−4^ C m^−2^ K at room temperature, enabling infrared sensing over the entire infrared region [33]. Ferroelectric LiNbO_3_ (LNO) materials are important functional materials for constructing integrated optics, nonlinear optics, and optoelectronic devices. Due to their high pyroelectric coefficient of −40 μC m^−2^ K^−1^, LNO materials exhibit a highly sensitive infrared response [172]. Heterostructures based on LNO and graphene materials can have a wide spectral response region (0.405 μm to 2 μm). 

A recently emerging class of ferroelectric materials for infrared sensing applications is that of the molecular perovskite materials, which possess infinite structural flexibility, such as metal-halide perovskites, metal formates, and metal-free molecules [173]. Perovskite ferroelectric (IA)_2_(EA)_2_Pb_3_Cl_10_, where IA and EA stand for isoamylammonium and ethylammonium, respectively, shows almost unchanged dielectric constants over a wide range of temperatures, exhibiting exceptional pyroelectricity [9]. The pyroelectric effect in (IA)_2_(EA)_2_Pb_3_Cl_10_ materials enables their high photoresponse to wavelengths up to 1.95 μm. Aside from their high pyroelectricity, some ferroelectric materials exhibit a narrow energy bandgap, which makes them ideal functional materials for photon-type and hybrid-type infrared optoelectronic sensors. For example, ferroelectric YMnO_3_ (YMO) materials with a hexagonal structure possess a narrow bandgap (of about 1.55 eV), thereby having the potential for infrared optoelectronic sensing based on their photovoltaic and photoconductive effects [174]. However, driven by a totally different geometric force, YMO materials exhibit a small remnant polarization (5 μC cm^−2^), leading to a weak photoresponse. Bi doping can greatly increase the ferroelectricity of YMO materials while maintaining an almost unchanged bandgap, therefore promoting their pyroelectric and photovoltaic responses to infrared light. Infrared optoelectronic sensors based on YMO materials show a wide response region with wavelengths up to at least 0.9 μm. Most oxide ferroelectric materials have bandgaps that are usually larger than 2 eV, causing a weak or absent photovoltaic current when responding to infrared light. To solve this problem, a +2 valence d^8^ cation substitution at the B site of ABO_3_-type perovskite ferroelectrics has been utilized to effectively decrease the bandgap. For instance, Ni^2+^-doped ferroelectric 0.9KNbO_3_-0.1(BaNi_1/2_Nb_1/2_O_3−δ_) exhibits a narrow direct bandgap of 1.39 eV, enabling infrared sensing by generating photovoltaic signals [175]. Moreover, Ni-modified ferroelectric lead lanthanum zirconate titanate (PLZT) materials exhibit two new absorption peaks in the range of 0.6–1.0 μm and 1.0–1.2 μm compared to pristine PLZT, which has a large bandgap of about 3.3 eV, allowing for infrared optoelectronic sensing [176]. Table 4 summarizes the key metrics of advanced infrared optoelectronic sensors constructed based on ferroelectric materials.

### 4.4. Organic Semiconductor Materials

Organic semiconductor materials are appealing candidates for constructing infrared optoelectronic sensors due to their intrinsic flexibility, light weight, low cost, scalability and ease of fabrication [8]. Additionally, organic semiconductor materials allow for cooling-free infrared sensing, thereby having great potential for future wearable devices. Significant efforts have been made to explore suitable organic semiconductors for high-performance infrared optoelectronic sensors. Currently, research on organic semiconductor materials for infrared optoelectronic sensors mainly focuses on developing narrow-bandgap organic polymers and small molecules.

To obtain narrow-bandgap polymer materials, a variety of methods have been developed, including donor–acceptor structures, chemical rigidification, quinone structures, and inductive and mesomeric effects. Of these methods, donor–acceptor structures and quinone structures are most used. Donor–acceptor-structured polymers can be fabricated by polymerizing donor and acceptor units. During the polymerization process, two new highest occupied molecular orbital (HOMO) energy levels, together with two new lowest unoccupied molecular orbital (LUMO) energy levels, are generated, forming organic polymers which have narrower bandgaps with higher HOMO and lower LUMO energy levels. Organic polymers with quinone structures can be synthesized by breaking the aromatic units in their backbone and converting the broken aromatic units into double-bond linkages to achieve narrower bandgaps.

The first narrow-bandgap organic polymer for infrared optoelectronic sensing was reported in 2007, which is a new kind of ester group-modified polythieno[3,4-b]thiophene (PTT) [182]. The absorption onset of the fabricated PTT films possesses a bandgap of 1.3 eV, making them well-suited for detecting infrared light with a wavelength less than about 0.95 μm. In 2009, poly(5,7-bis(4-decanyl-2-thienyl)-thieno(3,4-b)diathiazole-thiophene-2,5), with a narrow bandgap of approximately 0.8 eV, was developed for highly sensitive infrared sensing applications, extending the photoresponse region of organic polymer materials to about 1.45 μm [183]. An organic D-A copolymer PBBTPD composed of a donating dithienopyrrole group and a strong accepting benzobisthiadiazole unit shows an absorption onset at 2.5 μm and was presented in 2018, greatly extending the infrared spectral response range [184]. Since then, many other narrow-bandgap polymers have been synthesized for infrared optoelectronic sensing, such as polypyrrole nanoparticles, PTTQn(HD), PBTTT, diketopyrrolopyrrole DPP-based polymer PDPP-DTT, indanone-condensed thiadiazolo[3,4-g]quinoxaline-based polymer PBTTQCN-TT, and PDPP3T:PC_61_BM [185,186,187,188,189].

Compared to polymers, organic small molecules possess superior characteristics such as high carrier mobility, well-defined structures, and excellent reproducibility. A variety of narrow-bandgap small molecules have been developed for infrared sensing by utilizing various units within different molecular frameworks. Of these small molecules, phthalocyanines are the conventional materials used for constructing infrared optoelectronic sensors. The frequently utilized phthalocyanines include copper phthalocyanine, copper hexadecafluorophthalocyanine, lead phthalocyanine (PbPc), zinc phthalocyanine, phthalocycanine, vanadylphthalocyanine, tin phthalocyanine, and chloroaluminum phthalocyanine [190,191,192,193,194,195,196,197]. Another important kind of infrared-sensitive small molecules is the group of porphyrin-based compounds. For example, the porphyrin small molecule DHTBTEZP shows a good response in the near infrared region. More advanced infrared optoelectronic sensors based on organic materials are presented in Table 5.

### 4.5. Others

Aside from the abovementioned advanced materials, which have been extensively researched, some other materials have also been utilized to construct high-performance infrared optoelectronic sensors, such as mercury chalcogenides and organic–inorganic hybrid perovskite semiconductors. Mercury chalcogenides exhibit unique ultra-broad and tunable photoresponses across the near-infrared and mid-infrared regions, demonstrating an optoelectronic sensing performance comparable to commercial devices and particularly advantageous at high operating temperatures [204]. Among the various mercury chalcogenides, HgCdTe shows a broad tunability of its absorption spectrum (3–12 μm) and a long photocarrier lifetime (up to 1 μs), having remained the leading material for infrared detection for years. HgCdTe emerged as a photoconductive material with a performance comparable to that of InSb in 1959 [205]. Its bandgap can be conveniently adjusted by controlling the Hg to Cd ratio. Recently, HgTe nanocrystals have been proven to be promising materials capable of replacing the bandgap tunability of HgCdTe alloys, transitioning from mixed composition to quantum confinement. The bandgap of HgTe materials can be modulated from 1.5 eV to almost 0.02 eV [204]. Organic–inorganic hybrid perovskite semiconductors have been considered one of the most promising materials for infrared sensing applications due to their long excitation diffusion length, high charge carrier mobility, direct bandgap, and high absorption coefficient [206]. The performance of state-of-the-art infrared optoelectronic sensors based on diverse mercury chalcogenide quantum dots and organic–inorganic hybrid perovskite semiconductors is provided in Table 6.

## 5. Applications

Infrared optoelectronic sensors have become deeply integrated into modern technology and human society, with applications spanning image sensing, optical neuromorphic computing, logic operations, and health monitoring.

### 5.1. Image Sensing

**Figure 5:**
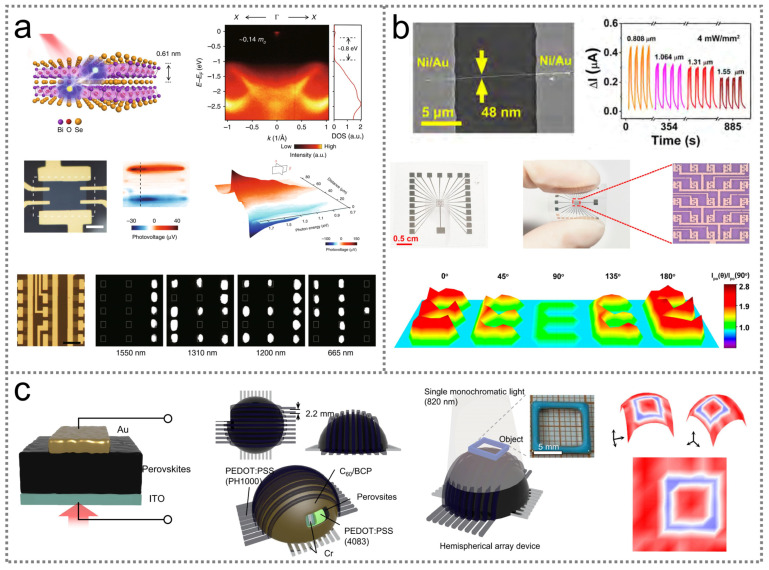
Infrared optoelectronic sensors for image sensing. (**a**) Infrared optoelectronic sensor based on Bi_2_O_2_Se for image sensing [246] (with permission from Springer Nature, 2018). (**b**) Device design and performance of an infrared polarimetric image sensor [149] (with permission from the American Chemical Society, 2022). (**c**) A hemispherical infrared optoelectronic sensor based on perovskites for wide-angle imaging sensing [247] (with permission from Springer Nature, 2022).

Imaging sensing is one of the most widespread applications of infrared optoelectronic sensors, and a large number of image devices based on different materials have been developed. For instance, a highly sensitive infrared optoelectronic image sensor has been developed utilizing a two-dimensional Bi_2_O_2_Se crystal exhibiting a high sensitivity of 65 AW^−1^ at 1.2 μm and an ultrafast response speed of about 1 ps at room temperature [246]. Figure 5a illustrates the schematic diagram and the performance of a single pixel of the Bi_2_O_2_Se infrared image sensor. The device demonstrates a broadband response with a spectrum ranging from visible to 1.7 μm. Scanning a laser beam over the sensor results in a photovoltage distribution, generating electrical signals with opposite polarities at its Bi_2_O_2_Se/metal interfaces, indicating that the electrical signals are primarily produced by photogenerated carrier separation at the symmetric Bi_2_O_2_Se/metal interfaces. Bi_2_O_2_Se infrared image sensors can be fabricated on a flexible mica substrate and consistently deliver a photocurrent even if undergoing a bending process with a strain of 1%. Moreover, these devices exhibit a non-attenuating photocurrent after more than 4 weeks, indicating their excellent stability in the ambient environment. When illuminated by lasers with different wavelengths (1.55 μm, 1.31 μm, 1.2 μm, and 0.655 μm), the photocurrent generated from a 3 × 5 multi-pixel Bi_2_O_2_Se sensor array can clearly show the laser beam’s distribution (Figure 5a).

Infrared polarimetric image sensors are capable of extending their detection of photoelectric signals from light wavelength and intensity to the light’s polarization vector, exhibiting great potential in the fields of remote image sensing, medical diagnosis, and environmental monitoring. Figure 5b exhibits an infrared polarimetric image sensor constructed utilizing sulfur-passivated GaSb nanowire arrays [149]. This 5 × 5 GaSb sensor array with interdigital electrodes is fabricated on a flexible PET substrate. One single pixel demonstrates a high responsivity of 9.39 × 10^2^ A W^−1^, an ultrahigh detectivity of 1.10 × 10^11^ Jones, a high dichroic ratio of 2.65, and a wide spectral response from 0.808 μm to 1.55 μm. With the assistance of a hollow mask “E”, linear polarization light with a wavelength of 1.55 μm can be illustrated on the GaSb sensor array within the “E” area. The output three-dimensional images show a clear “E” pattern, with polarization angles of 0° and 180° stronger than those of 45°, 90°, and 135°, demonstrating its significant near-infrared polarization imaging ability.

In addition to the abovementioned flat image sensors, infrared optoelectronic sensors can also be designed in a hemispherical shape for wide-angle imaging applications, as illustrated in Figure 5c [247]. A hemispherical image sensor with 9 × 9 pixels can be constructed based on photosensitive phenylethylammonium/formamidinium lead halide perovskite materials using a spray-coating method. The device can capture images at various wavelengths, with a wide incident light angle of 180°.

### 5.2. Optical Neuromorphic Computing

**Figure 6:**
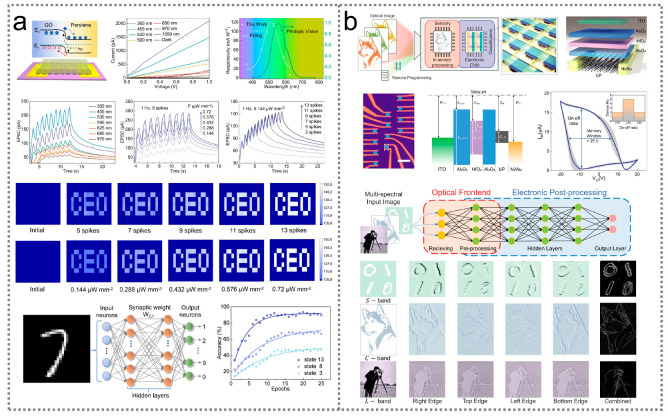
Infrared optoelectronic sensor for neuromorphic computing. (**a**) Device design, photonic synaptic characteristics, and image memorization and recognition capability of an infrared optoelectronic sensor based on a perylene/graphene oxide heterostructure [248] (with permission from Springer Nature, 2022). (**b**) Device configuration, current-voltage characteristics, and in-sensor computing performance of an infrared optoelectronic sensor based on black phosphorus materials [249] (with permission from Springer Nature, 2022).

Neuromorphic optoelectronic sensors, which utilize artificial photosensitive synapses, are capable of emulating biological nervous systems, with in-memory sensing and computing abilities. Based on a planar heterostructure composed of perylene and graphene oxide, optoelectronic sensors exhibit a broadband photoperception range from 0.365 μm to 1.55 μm and an ultrahigh specific detectivity of 3.1 × 10^13^ Jones [248]. Figure 6a exhibits the schematic diagram and performance of the sensor, where a type-II band structure is formed at the perylene/graphene oxide interface due to the energy level mismatch in the perylene and graphene oxide materials. The perylene/graphene oxide heterostructure can effectively absorb photons and separate photogenerated electron–hole pairs for photoelectric signal generation. Upon illumination, the sensor is able to emulate visual perception and diverse synaptic plasticity, including biological neurons, with the features of an excitatory postsynaptic current, spike-intensity-dependent plasticity, spike-number-dependent plasticity, and short-/long-term memories. When a pattern image is fed into a 10 × 10 perylene/graphene oxide array, the weighted graphs of the learning patterns become more and more distinct with the light pulse number, facilitating image recognition. By utilizing the sensor array as a neuron, the accuracy of an artificial neural network for image classification can reach 85%. Additionally, infrared optoelectronic sensors can also be utilized for edge computing (in-sensor computing), significantly reducing communication latency and energy consumption for artificial vision in distributed systems and robotic devices. As illustrated in Figure 6b, a BP-based programmable phototransistor optoelectronic sensor is capable of being programmed with 5-bit precision to perform an in-sensor convolutional neural network, with a high accuracy of 92%, by electrically and optically modulating the stored charges in its gate dielectrics [249].

### 5.3. Logic Operation

**Figure 7:**
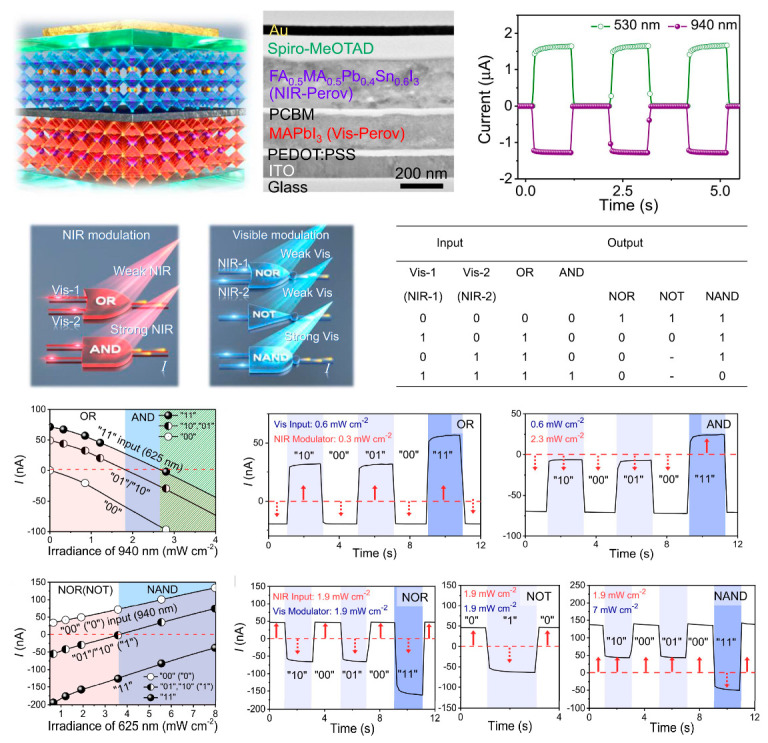
Device design and outputs of a perovskite infrared optoelectronic sensor for logic operations. The device can achieve the five basic logic operations of “OR”, “AND”, “NAND”, “NOR”, and “NOT” by controlling the wavelength and intensity of its incident lights [250] (with permission from Springer Nature, 2022).

The growing demand for a wide range of data processing has driven interest toward optoelectronic logic gate platforms because of their broad bandwidth and fast data transmission. An optoelectronic sensor based on a back-to-back p+-i-n-p-p+ diode structure exhibits a bipolar spectral photoresponse to visible and infrared light [250]. As illustrated in Figure 7, the sensor consists of vertically stacked low-bandgap (1.21 eV) perovskite FA_0.5_MA_0.5_Pb_0.4_Sn_0.6_I_3_ for infrared photon adsorption, and a large-bandgap (1.67 eV) perovskite MAPbI_3_ for visible photon adsorption. When illuminated by visible and infrared light, the sensor generates currents with positive and negative polarities, respectively, paving the way for optical logic gate operations. By controlling the wavelength and intensity of the incident light, a single sensor can achieve five basic logic operations “OR”, “AND”, “NAND”, “NOR”, and “NOT”. For instance, the sensor executes an “AND” operation when illuminated with visible light (0.625 μm at 0.6 mW cm^−2^) combined with intensity-varying near-infrared light (0.94 μm).

### 5.4. Health Monitoring

**Figure 8:**
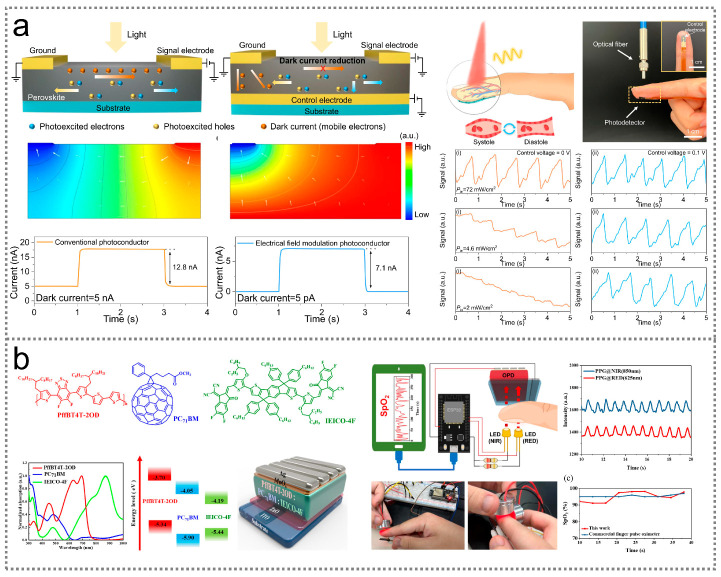
Infrared optoelectronic sensors for health monitoring. (**a**) Device configuration, working mechanism, and outputs of a perovskite infrared optoelectronic sensor for detecting blood pulse frequency [251] (with permission from Springer Nature, 2023). (**b**) Device structure and outputs of an organic infrared optoelectronic sensor for monitoring blood oxygen saturation [252] (with permission from the American Chemical Society, 2023).

Infrared optoelectronic sensors provide an effective approach to monitoring health conditions, particularly blood pulse frequency and blood oxygen saturation (SpO_2_). Figure 8a shows a metal halide perovskites-based flexible optoelectronic sensor, which can be utilized for blood pulse signal detection based on photoplethysmography [251]. The basic working principle of the sensor can be described as follows: When light with a wavelength of 0.8 μm is incident on the fingertip, a part of the light is absorbed, reflected, and scattered by human tissues, and then transmits through the finger to enter the flexible optoelectronic sensor. During this process, the light intensity measured by the sensor fluctuates with the variations in the volume of the blood vessels, induced by the heartbeat. The contraction of the heart leads to an increase in the blood volume in the vessels, thereby decreasing the detectable light’s intensity. Conversely, the diastole of the heart results in a decreased blood volume in the vessels, hence increasing the detectable light’s intensity. In this way, the heart rate can be extracted from the generated electrical signals of the sensor for cardiopulmonary function evaluations. When the light is turned on, reproducible electrical signals can be observed due to the periodic contraction and diastole of the human heart, from which a patient’s blood pulse frequency can be derived according to the electrical signal’s frequency. An infrared optoelectronic sensor for SpO_2_ monitoring is illustrated in Figure 8b [252]. This optoelectronic sensor is fabricated based on a PffBT4T-2OD:PC_71_BM heterostructure, which exhibits a high detectivity of 7.2 × 10^12^ Jones at 0.85 μm. According to the Beer–Lambert law, SpO_2_ can be derived from the difference in transmission at specific wavelengths of light utilizing the equation [252,253]

(11) $$S_{p}O_{2}=\frac{\varepsilon_{\mathrm{Hb}}\left(\lambda_{625}\right)-\varepsilon_{\mathrm{Hb}}\left(\lambda_{850}\right)R_{\mathrm{os}}}{\left[\varepsilon_{\mathrm{Hb}}\left(\lambda_{625}\right)-\varepsilon_{{\mathrm{HbO}}_{2}}\left(\lambda_{625}\right)\right]+\left[\varepsilon_{{\mathrm{HbO}}_{2}}\left(\lambda_{850}\right)-\varepsilon_{\mathrm{Hb}}\left(\lambda_{850}\right)R_{\mathrm{os}}\right]}$$

where *ε*_Hb_ and *ε*_HbO2_ stand for the molar extinction coefficients of oxyhemoglobin and deoxyhemoglobin, respectively. *R*_os_ can be obtained from the ratio of the pulsatile (AC) and stationary (DC) parts of the photoplethysmogram signals utilizing the formula [252,253]

(12) $$R_{\mathrm{OS}}=\frac{AC_{850}/DC_{850}}{AC_{625}/DC_{625}}$$

Due to its broadband photoresponse, the PffBT4T-2OD:PC_71_BM optoelectronic sensor exhibits distinct photocurrent signals under the illumination of both 0.625 μm and 0.85 μm wavelengths, achieving SpO_2_ detection.

### 5.5. Others

**Figure 9:**
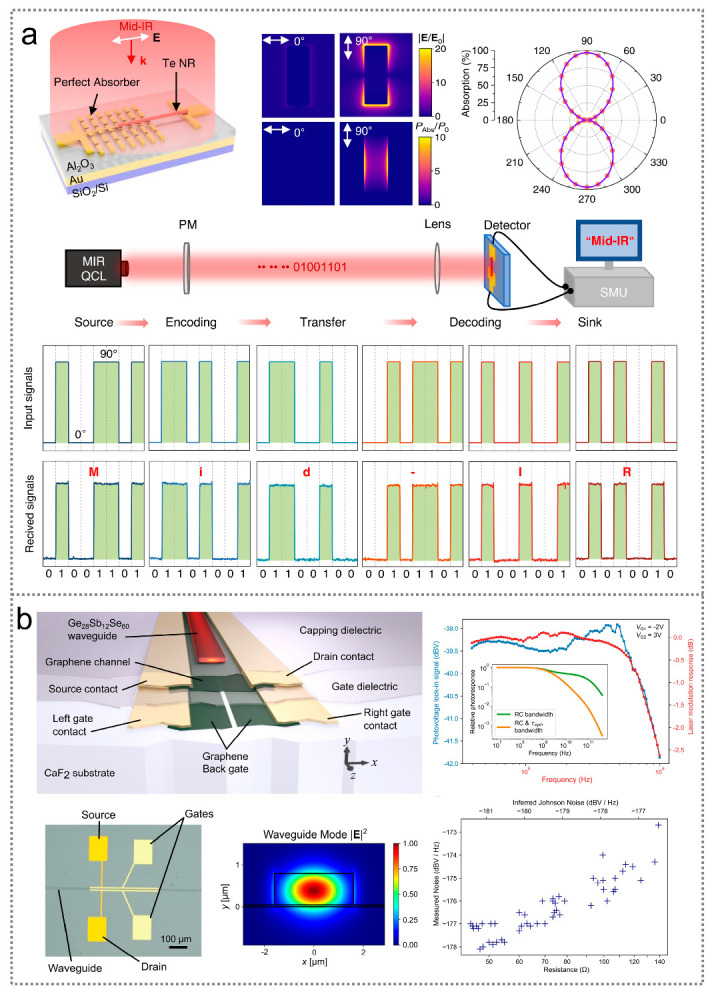
Infrared optoelectronic sensors for optical communication and gas sensing. (**a**) Device design and polarization-coded communication process of a polarization-sensitive infrared optoelectronic sensor [254] (with permission from Springer Nature, 2023). (**b**) Schematic diagram, frequency response, and measured noise spectral density of a graphene-based infrared optoelectronic sensor for gas sensing [255] (with permission from Springer Nature, 2022).

In addition to the aforementioned applications, infrared optoelectronic sensors also demonstrate potential applications in optical communication and gas sensing. Figure 9a illustrates a photothermoelectric infrared optoelectronic sensor used for optical communication applications [254]. The sensor is fabricated utilizing tellurium nanoribbons. By converting its polarization-sensitive absorption into a large temperature gradient with the assistance of the finite-size effects of perfect plasmonic absorbers, the sensor shows advantages including a high responsivity (410 V W^−1^) and ultrahigh polarization ratio (2.5 × 10^4^) that make it well suited for polarization-coded communication. During the communication process, mid-infrared light can first be converted into American Standard Code for Information Interchange (ASCII) codes by varying the polarization angle, with 0° for “0” and 90° for “1”, respectively. In this way, the input signals can be encoded by the polarization-sensitive sensor and finally fed into a terminal computer. The received signals match well with the input signals, demonstrating the capability of the sensor for information transmission. Figure 9b illustrates an infrared optoelectronic sensor for gas sensing applications [255]. The device is composed of a single-mode Ge_28_Sb_12_Se_60_ waveguide, a graphene channel, split graphene back-gates, and HfO_2_ gate dielectrics. The graphene back-gates can be utilized to electrostatically create a p-n junction along the center of the graphene channel. The sensor enables NO detection at concentrations comparable to the recommended exposure limit of 25 ppm, which pushes forward the development of gas sensing microsystems.

## 6. Conclusions and Prospects

In this review, we summarized the current status of infrared optoelectronic sensors. Based on their working mechanisms, these sensors can be roughly classified into three categories: photon, photothermal, and hybrid sensors. Two-dimensional semimetals and semiconductors, group III–V semiconductors, ferroelectric materials, and organic semiconductors exhibit unique advantages in infrared optoelectronic sensing. For instance, 2D semimetals and semiconductors are suitable for high-speed photodetection due to their high carrier mobility, and ferroelectric materials have the potential to simultaneously utilize pyroelectric and photovoltaic effects for an enhanced photoresponse. Embedded deeply in modern technology and human society, infrared optoelectronic sensors show promise in diverse applications, including image sensing, neuromorphic computing, logic operations, optical communication, health monitoring, and gas sensing.

While plenty of advancements have been made in exploring infrared optoelectronic sensors, several challenges remain in this field. (1) The response speed of most existing infrared optoelectronic sensors is in the order of microseconds, which is insufficient for capturing rapidly changing light. (2) Comprehensive characterizations of infrared optoelectronic sensors, including their response range, responsivity, specific detectivity, response time, NEP, LDR, and EQE, are needed for the further optimization of these devices. (3) Although some infrared optoelectronic sensors have been utilized for health monitoring applications, research on their biocompatibility is lacking.

Considering the growing demand for high-performance infrared optoelectronic sensors, various research directions could be pursued to advance the development of infrared optoelectronic sensors in the near future. (1) Most 2D materials for infrared optoelectronic sensors are fabricated based on small-size mechanically exfoliated flakes, so it is essential to develop techniques for their scalable production. The most promising methods to achieve large-scale 2D materials might be CVD and molecular beam epitaxy. (2) Infrared optoelectronic sensors based on narrow-bandgap semiconductors usually exhibit unstable performances due to variations in ambient temperature, hence developing temperature-insensitive devices is of great importance. Micro-constant temperature systems which can be integrated into infrared optoelectronic sensors can help solve this problem. (3) The development of optical logic operations and optical communication pose new challenges for high-frequency devices; therefore, ultrafast infrared optoelectronic sensors are desirable. To achieve this, materials with ultrahigh carrier mobility and homo-/heterostructures with strong built-in electric fields are required. (4) The investigation of biocompatible infrared optoelectronic sensors is promising for future healthcare. This might be realized using non-toxic organic semiconductors. (5) In optical neuromorphic computing applications, the development of photosynapses with a long memory, for weight storage, is essential. Carrier trapping using device interfaces or ferroelectric polarization are promising for achieving this goal. Overall, the development of infrared optoelectronic sensors will contribute to advancements in daily life, industry, and the medical field, and more efforts are needed to further improve their performance.

## Figures and Tables

**Table 2:** Performance of advanced infrared optoelectronic sensors based on 2D semiconductors.

Active Material	Spectral Range (μm)	Responsivity (A W^−1^)	Specific Detectivity (Jones)	Response Speed (μs)		Ref.
				Rise Time	Fall Time	
Graphene	1.55–11	0.28–38	1 × 10^9^	-	-	[102]
Graphene	5–20	7.62 × 10^6^	6.24 × 10^13^			[103]
Antenna/graphene	5.5–7.5	0.27	-	9.5 × 10^−3^	-	[104]
Graphene/SnS_2_	0.365–2.24	2	1.8 × 10^10^	-	-	[105]
Graphene/Si	0.375–3.8	0.1	-	1	-	[106]
Graphene/Si	1.1–4.0	0.003–0.011	1.6 × 10^11^	0.02–0.03	-	[107]
Tellurium	0.52–3.0	1.36 × 10^3^	1.15 × 10^10^	48.7–62.7	62.7–78.0	[108]
Tellurium	0.83–1.55	6.65 × 10^3^	4.68 × 10^8^	31.7	25.5	[109]
Tellurium	0.635–1.55	9.38	1.19 × 10^10^	70	-	[110]
BP	0.52–1.45	1.06	1 × 10^11^	-	-	[40]
BP/MoS_2_	2.3–3.5	-	1.2 × 10^10^	-	-	[111]
B-AsP	0.52–4.6	0.023	2.7 × 10^10^	0.4	0.6	[112]
B-AsP	0.98–1.55	1.02 × 10^4^	-	8.2 × 10^4^	9.2 × 10^4^	[113]
InSe	0.976	0.38	-	4.1 × 10^−3^	-	[114]
InSe	0.5–1.45	56	5.70 × 10^10^	0.17 × 10^6^	-	[115]
InSe/PdSe_2_	≤1.65	58.8	1 × 10^10^	7.2 × 10^4^	1.8 × 10^5^	[116]
α-In_2_Se_3_	0.325–1.8	1.081 × 10^3^	5.61 × 10^9^	8 × 10^3^	-	[117]
α-In_2_Se_3_/Si	0.405–0.98	0.56	1.6 × 10^13^	43	-	[118]
β-In_2_Se_3_/Si	0.265–1.3	6.4	4.3 × 10^10^	2.2	-	[119]
In_2_Se_3_	0.59–0.94	5.6	7 × 10^10^	1.4 × 10^5^	2.5 × 10^4^	[120]
SnS	0.405–0.808	1.62 × 10^4^	-	3.84 × 10^4^	3.98 × 10^4^	[121]
SnSe	0.532–1.064	1.4 × 10^−3^	2.36 × 10^8^	5 × 10^3^	2.8 × 10^4^	[122]
SnSe	0.44–0.85	78.6	9 × 10^11^	2.3 × 10^5^	2.7 × 10^5^	[123]
SnSe/InSe	0.405–0.808	0.35	5.8 × 10^9^	2.6 × 10^5^	1.7 × 10^5^	[124]
SnSe_2_	0.45–1.55	0.761	1.03 × 10^12^	2.13 × 10^4^	2.04 × 10^4^	[125]
SnSe_2_/MoSe_2_	0.39–1.088	7.09	6.44 × 10^12^	2.1 × 10^5^	-	[126]
MoS_2_	0.532–1.064	1.65 × 10^4^	-	4.63 × 10^5^	3.15 × 10^5^	[127]
MoS_2_	0.473–2.712	0.0475	1.26 × 10^7^	1 × 10^4^	1.6 × 10^4^	[128]
MoSe_2_/GaAs	0.405–0.808	5.25	1.13 × 10^13^	2.9	1.8	[129]
MoSe_2_	0.52–0.94	2.25	10^10^	490	495	[130]
MoTe_2_/MoS_2_	0.4–1.48	9.74	-	42	-	[131]
WS_2_/Ge	0.2–4.6	0.634	4.3 × 10^11^	9.8	12.7	[132]
WSe_2_/Si	0.265–1.55	0.689	1.59 × 10^12^	0.215	-	[133]
WTe_2_	0.32–1.2	8.5 × 10^−4^	1.23 × 10^8^	3.2 × 10^5^	3.7 × 10^5^	[134]
Bi_2_Se_3_/Si	≤0.808	24.28	4.39 × 10^12^	2.5	5.5	[135]
Sb_2_Se_3_/Si	0.43–0.98	-	-	1.6 × 10^5^	2.8 × 10^5^	[136]
Sb_2_Te_3_/Si	0.405–1.55	0.15	1.65 × 10^14^	98	133	[137]
ReS_2_/perovskite	0.532–2.0	2.2	1.8 × 10^14^	443	720	[138]
ReSe_2_/PtSe_2_	0.405–0.98	0.153	7.72 × 10^11^	-	-	[139]
PtSe_2_/Ge	≤1.55	0.766	1.1 × 10^11^	54.9	56.6	[140]
PtTe_2_	0.2–0.98	0.406	3.62 × 10^12^	7.51	36.7	[141]
Ta_2_NiSe_5_/WSe_2_	0.532–2.2	10^3^	-	1	-	[142]
HgCdTe/BP	0.637–4.3	0.193	7.93 × 10^10^	150	110	[143]
SnP_2_Se_6_	0.78–1.56	10^3^	5.1 × 10^10^	412	-	[144]

**Table 3:** Performance of advanced infrared optoelectronic sensors based on group III–V semiconductors.

Active Material	Spectral Range (μm)	Responsivity (A W^−1^)	Specific Detectivity (Jones)	Response Speed (μs)		Ref.
				Rise Time	Fall Time	
InGaAs	0.6–1.7	0.53	5.18 × 10^11^	-	50.8	[154]
InGaAs	1.2–1.7	0.2	-	-	-	[151]
In_0.51_Ga_0.49_As	≤1.55	7300	4.2 × 10^10^	480	810	[155]
InSb	0.637–4.3	311.5	9.8 × 10^9^	4.2 × 10^6^	5.5 × 10^6^	[156]
InAs	2.0–3.5	0.44	1.25 × 10^10^	25	24	[157]
InAs/SnBr_2_	0.98–1.1	0.36	-	13.5	9	[158]
GaAs_1–x_Sb_x_/InAs	0.488–1.8	0.12	-	4.5 × 10^3^	-	[152]
InAs/GaAs	1–3.5	-	2 × 10^8^	-	-	[159]
InSb	≤5.3	-	8.8 × 10^9^	-	-	[160]
GaSb/GeS	0.405–1.31	0.061	6.8 × 10^11^	2 × 10^6^	1.2 × 10^4^	[161]
In_x_Ga_1−x_Sb	0.532–1.55	6 × 10^3^	3.7 × 10^9^	38	53	[162]
InGaAsSb	≤2.2	2.9 × 10^−6^	7.4 × 10^11^	-	-	[163]
In_0.28_Ga_0.72_Sb	≤1.55	1.52 × 10^3^	-	13	16	[164]
InGaAs	≤1.6	6.5 × 10^3^	-	7 × 10^4^	2.8 × 10^5^	[148]
GaAsSb	≤1.31	1.7 × 10^3^	-	6 × 10^4^	-	[155]
InGaAs	≤1.55	7.3 × 10^3^	4.2 × 10^10^	480	-	[165]
GaSb	≤1.55	77.3	1.14 × 10^10^	-	-	[166]
In-rich InGaAs	≤1.55	5.75	-	-	-	[167]
GaAsSb/GaAs	≤0.9	110	1.1 × 10^14^	-	-	[168]
GaN/AlN	≤1.55	1.1	-	-	-	[153]

**Table 4:** Performance of advanced infrared optoelectronic sensors based on ferroelectric materials.

Active Material	Spectral Range (μm)	Responsivity (A W^−1^)	Specific Detectivity (Jones)	Response Speed (μs)		Ref.
				Rise Time	Fall Time	
(IA)_2_(EA)_2_Pb_3_Cl_10_	0.266–1.95	-	-	-	-	[9]
PMN-PT	0.375–118.8	1.64 × 10^−8^	-	-	-	[33]
YMO	0.365–0.9	0.6	2.5 × 10^12^	400	300	[174]
PLZT	≤1.3	1.64 × 10^−7^	4.05 × 10^7^	-	-	[176]
YMO/graphene	6–10	-	1.14 × 10^5^	1.3 × 10^6^	-	[177]
LNO	0.405–2	2.92 × 10^6^	8.6 × 10^14^	2.3 × 10^4^	2.3 × 10^4^	[178]
BZT-BCT	0.3–2.5	5.32 × 10^−8^	1.49 × 10^5^	-	-	[179]
LuMnO_3_	≤0.9	0.39	6.89 × 10^11^	1.44 × 10^4^	1.64 × 10^4^	[180]
BiFeO_3_	0.005–0.808	1.8 × 10^3^	-	6.97 × 10^3^	1.2 × 10^3^	[181]

**Table 5:** Performance of advanced infrared optoelectronic sensors based on organic semiconductors.

Active Material	Spectral Range (μm)	Responsivity (A W^−1^)	Specific Detectivity (Jones)	Response Speed (μs)		Ref.
				Rise Time	Fall Time	
PPy-NPs	0.8–2	1.3	-	130	203	[186]
PBTTT:PC_71_BM	0.75–1	0.05	-	-	-	[187]
PBTTT:PC_61_BM	0.775–1.075	-	-	-	-	[187]
PDPP-DTT	0.808–1.55	2 × 10^3^	-	1.8 × 10^5^	1.5 × 10^5^	[188]
PDPP3T:PC_61_BM	0.405–0.85	-	7.8 × 10^13^	2.24 × 10^4^	6.8 × 10^3^	[189]
TPBi:PDPP3T:PC_61_BM	0.405–0.85	-	4.49 × 10^14^	1.09 × 10^4^	4.5 × 10^3^	[189]
PDPP3T:PS:PC_61_BM	0.405–0.85	-	1.52 × 10^14^	2.31 × 10^4^	9.4 × 10^3^	[189]
TPBi:PDPP3T:PS:PC_61_BM	0.405–0.85	-	5.43 × 10^14^	5.3 × 10^3^	1.8 × 10^3^	[189]
η-F_16_CuPc	0.589–0.94	-	-	4.48 × 10^5^	4.48 × 10^5^	[191]
PbPc	0.9–1	0.035	-	-	-	[192]
ZnMe2Pc	0.698–0.79	0.013	-	-	-	[193]
8OH_2_Pc	≤0.77	-	2.1 × 10^12^	-	-	[194]
SnPc	0.405–0.98	7.2 × 10^−4^	6.98 × 10^9^	-	-	[196]
ClAlPc	≤0.78	-	5.8 × 10^12^	0.75	0.7	[197]
CDT-TQ:PC_71_BM	0.4–1.4	0.1	7.8 × 10^9^	167	-	[198]
CO1-4Cl	0.92–0.96	0.5	3.1 × 10^13^	-	-	[199]
PBBTCD	0.8–1.2	4 × 10^−4^	-	51	55	[200]
TQ-T:IEICO-4F	0.35–1.8	8.4 × 10^−6^	10^10^	-	-	[201]
BDP-OMe:C_60_	0.3–0.9	-	10^13^	-	-	[202]
CPDT-TQ:PC_71_BM	0.9–1.31	-	5 × 10^10^	-	-	[203]

**Table 6:** Performance of advanced infrared optoelectronic sensors based on mercury chalcogenide quantum dots and organic–inorganic hybrid perovskite semiconductors.

	Active Material	Spectral Range (μm)	Responsivity (A W^−1^)	Specific Detectivity (Jones)	Response Speed (μs)		Ref.
					Rise Time	Fall Time	
Mercury chalcogenide	HgCdTe/graphene	4	2.5	2 × 10^10^	0.013	-	[207]
	HgTe	≤2.6	1	>10^10^	-	-	[208]
	HgTe	≤2.5	0.25	1.5 × 10^10^	1	-	[209]
	HgTe	≤2.3	0.023	3.2 × 10^6^	-	-	[210]
	HgTe	≤4.8	0.23	5.4 × 10^10^	0.9	2	[211]
	HgTe	≤2.5	10^3^	10^12^	20	-	[212]
	HgTe	≤4	0.7	2 × 10^10^	11	-	[213]
	HgTe	≤4	0.32	7 × 10^10^	0.07	-	[214]
	HgTe-P3HT	2.5	1	>10^11^	-	1.5	[215]
	HgTe-graphene	≤3	150	6 × 10^8^	400	700	[216]
	HgTe p-n junction	≤2.5	2 × 10^−3^	-	-	3 × 10^−3^	[217]
	HgTe/Ag_2_Te	≤3–5	1.62	4 × 10^11^	<1	-	[218]
	HgTe/HgTe	≤2.5	0.02	3 × 10^9^	-	0.37	[219]
	Bi_2_Se_3_/HgTe/Ag_2_Te	≤2.5	0.22	7.5 × 10^10^	-	-	[220]
	Bi_2_Se_3_/HgTe/Ag_2_Te	1.5–2.1	0.2	>10^10^	0.12	-	[221]
	SnO_2_/HgTe/Ag_2_Te	≤2	0.3	5 × 10^10^	0.3	-	[222]
	CdSe_2_/HgTe/Ag_2_Te	≤2	0.8	9 × 10^10^	0.17	-	[223]
	HgSe	≤4.2	0.077	1.7 × 10^9^	-	-	[224]
	HgSe/HgTe	≤4	0.003	10^9^	0.17	0.15	[225]
Hybrid perovskite	MAPbI_3_/Gd-ZnO	0.25–1.357	0.22	9.3 × 10^9^	4 × 10^5^	5 × 10^5^	[226]
	MAPbI_x_Cl_3−x_/Si	0.3–1.15	0.87	6 × 10^12^	5 × 10^4^	1.5 × 10^5^	[227]
	MAPbI_3_/CuO	0.35–1.05	0.562	2.15 × 10^13^	2 × 10^5^	2 × 10^5^	[228]
	MAPbI_3_/PbSe	0.3–2.6	0.628	2.59 × 10^12^	4	32	[229]
	MAPbI_3_/CuInSe_2_	0.3–1.1	0.15	7.7 × 10^11^	0.277	0.277	[230]
	MAPbI_2.5_Br_0.5_/PbS	0.4–1.4	0.99	4 × 10^12^	<10	<10	[231]
	MAPbI_3_/PDPPTDTPT	0.35–1.05	-	1 × 10^11^	6.1 × 10^−3^	6.1 × 10^−3^	[232]
	MAPbI_3_	0.4–1	4	-	39	1.9	[233]
	FA_0.85_Cs_0.15_PbI_3_/Bi_2_Se_3_	0.3–1	1.02	2.08 × 10^12^	16	14	[234]
	MAPbI_3_/SnPc	0.3–1	0.72 × 10^−3^	6.98 × 10^9^	390	530	[196]
	MAPbI_3_/MoS_2_	0.5–0.85	110	7.93 × 10^7^	6.17 × 10^6^	4.5 × 10^6^	[235]
	MAPbI_3_/PbSe	0.3–1.5	0.7	7 × 10^7^	2.5 × 10^3^	3 × 10^3^	[236]
	MAPbI_3−x_Cl_x_/PbS	0.3–1.5	0.35	9 × 10^10^	250	500	[237]
	MAPbI_3−x_(SCN)_x_/Si	0.35–1.1	13	1 × 10^13^	22.2	17.6	[238]
	MASnI_3_	0.2–1	0.47	8.8 × 10^10^	1.5 × 10^6^	4 × 10^5^	[239]
	FASnI_3_	0.3–1	2 × 10^3^	3.2 × 10^12^	-	-	[240]
	FA_0.85_Cs_0.15_Sn_0.5_Pb_0.5_I	0.6–1	0.53	6 × 10^12^	5.83 × 10^−2^	0.86	[241]
	(FASnI_3_)_0.6_(MAPbI_3_)_0.4_	0.3–1	0.4	1.1 × 10^12^	6.9	9.1	[242]
	MA_0.5_FA_0.5_Pb_0.5_Sn_0.5_I_3_	0.35–1	>0.2	>10^12^	-	-	[243]
	MASn_x_Pb_1−x_I_3_	0.3–1.1	0.2	>10^11^	0.09	2.27	[244]
	(CsPbI_3_)_0.05_(FAPbI_3_)_1−x_	0.405–0.81	84.77	3.22 × 10^12^	2.46 × 10^4^	1.47 × 10^4^	[245]

Notes: “MA” stands for methylammonium CH_3_NH_3_. “FA” stands for formamidinium CHNH_3_.
